# Institutionalization for good governance to reach sustainable health development: a framework analysis

**DOI:** 10.1186/s12992-023-01009-5

**Published:** 2024-01-02

**Authors:** Hajar Haghighi, Amirhossein Takian

**Affiliations:** 1 Department of Health Management, Policy & Economics, School of Public Health, Tehran University of Medical Sciences, Tehran, Iran; 2 Department of Global Health & Public Policy, School of Public Health, Tehran University of Medical Sciences, Poursina Avenue, Qods Street, Enqelab Square, Tehran, Iran; 3 Health Equity Research Centre (HERC), Tehran University of Medical Sciences, Tehran, Iran

**Keywords:** Institutionalization, Good governance, Sustainable development, Health, Framework

## Abstract

**Background:**

This article explores the concept of institutionalization, which is the process of transforming ideas into programs and automating actions, in the context of health system governance and sustainable development. Institutionalization is a key mechanism for creating accountable and transparent institutions, which are essential for achieving health system resilience and sustainability. This study identifies the components and dimensions of institutionalization in the health system and its relationship with good governance and sustainable health development.

**Main text:**

We applied a scoping review method in five steps. First, we formulated a question for our research. Then, we concluded a comprehensive literature search in five electronic databases for identifying relevant studies. This review has two phases: identifying the concept of institutional approach and its components in health system, and its relationship with good governance to reach Sustainable Health Development (SHD). The third step was study selection, and the 1st author performed data abstraction. The key issues which are identified in our review, related to the concepts of SDH, its goals, pillars and principles; positive peace; good governance; components of institutional approach components, and their relations. Finally, we summarized and organized our findings in a format of a proposed conceptual framework, to underpin the role of institutionalization in the health system to achieve sustainable development.

**Conclusion:**

Institutionalization is a key concept for achieving positive peace and good governance, which requires meaningful involvement of leaders, politicians, civil society, and public participation. It also depends on the conditions of justice, human rights, transparency, accountability and rule of law. In the wake of COVID-19, institutionalization is more crucial than ever for advancing sustainable development, especially in the context of low and middle-income countries (LMICs).

## Background

Institutionalization is described as the process in which assumed ideas are transformed into operational programs, that are accepted as effective ways to achieve the agreed objectives. It is a form of automation; when ideas and workflows become institutionalized, where they become entangled in formal organizational structures to automate actions. The more an idea is institutionalized, the less we need to think and act independently and reflectively [1, 2]. Institutionalization involves the processes by which social processes, obligations, or actualities come to take on a rule like status in social thought and action [3]. Further, institutionalization has been recognized as a prerequisite mechanism to establish good governance and the key for appropriate policy development [4], which is “the most important factor in eradicating poverty and promoting development’ [5].

In the context of Sustainable Development Goals’ (SDGs), SDG 16 is about peace, justice, and strong institutions. In particular, SDG 16.6 is dedicated to developing effective, accountable and transparent institutions at all levels. It emphasizes the importance of creating effective, accountable, and transparent institutions to reach more cooperative and sustainable societies as well as health systems [6]. Health is the only area of social policy, which is a prerequisite, output and indicator of a sustainable society simultaneously, and have to be accepted as a universal value and a common social and political goal for all human beings [7]. Numerous threats have led to rapid and unpredictable changes to sustainable health [8]. Conventionally, governments are responsible to improve citizens’ health and well-being and achieve sustainable and resilient health systems. In this regard, tailored institutional arrangements are essential to maintain and improve health systems in a very dynamic and complex global context [5, 6]. Stewardship is one of the key functions of health systems, which refers to the role of governments and other actors in setting the vision, direction, and goals of the system, as well as ensuring its accountability, responsiveness, and efficiency. It involves the establishment and enforcement of rules, norms, and standards that guide the behavior and performance of the system and its actors [9] Institutionalization is the process and outcome of creating, maintaining, and reinforcing these rules, norms, and standards, so that they become accepted and taken for granted as part of the system. Therefore, stewardship and institutionalization are closely related and mutually reinforcing concepts, which can contribute to the achievement of good governance and sustainable health development [10].

A meaningful and functional institutional arrangement in the context of health system is fundamental to ensure appropriate policy generation and adopt routinization of various healthcare functions, which might become standard practice ultimately [11]. Most notably, institutionalization renders customized incorporation of ideas and workflows into organizational structures, which may benefit policy makers and mangers for timely and integrated actions in any situation, including emergencies. In other words, institutionalization may facilitate resiliency and coherence among various governmental sectors, which is crucial for integrated policymaking and sustainability [1]. The capacity of stakeholders, the institutions and community representatives to prepare for and effectively respond to crises [12], in line with the institutional approach, all have strong relationship with health system resiliency [13].

Despite its wide recognition in the context of sustainable development and good governance, institutional approach has not been sufficiently developed in health system governance. This might be due to tiny evidence and practical case studies to identify the nature and dimensions of institutional approach in many health systems, particularly in low and middle-income countries (LMICs) [14, 15]. Therefore, developing a functional, meaningful, and customized framework is essential to nurture institutionalization into the context of health system in any setting. Using a scoping review approach, this article identifies the concept of institutional approach and its components in the health system, its relationship with good governance, and its application to reach sustainable health development (SHD).

## Main text

### Methods

This is an exploratory qualitative study that was carried out during 2022–2023 in the health system setting of Iran. We used Arksey and O’Malley’s scoping review approach [16, 17] to explore the breadth of knowledge and practice in the emerging field of institutionalization. Scoping review is a valuable method to explore the range of knowledge and practice, when the domain is unclear or has diverse methodological and conceptual attributes [18]. By including both qualitative and quantitative studies, scoping review allows mapping different types of evidence, which is particularly valuable when inadequate quantitative evidence is available [19]. Ideally, the scoping review framework include all the elements that are explained here in more depth and with some important points to consider [20]. Below, we describe the “five stages framework” suggested by Arksey and O’Malley [16].

#### Framework stage 1: identifying a research question

We focused on the following research query: what is known from the existing literature about the concept of institutional approach and its components in the health system? Our precise research questions were: 1) what is the relationship between establishing institutional approach and reaching good governance in any health system? And 2), how might this lead to achieving SHD?

#### Framework stage 2: identifying relevant studies

This review has two phases: identifying the concept of institutional approach and its components in health system, and its relationship with good governance to reach SHD. We searched relevant literature to identify appropriate keywords and components of each context. Consequently, five relevant databases (PubMed, Web of Science, Scopus, EMBASE and Google Scholar) were searched for related published studies by using identified keywords related to institutionalization, good governance and sustainable development. A summary of our adopted two-steps search strategy is illustrated in Table 1.

**Table 1:** Search strategies in five databases

Data Base	Search strategy	
	The first phase	The second phase
**PubMed**	PubMed = ((((((“health system“[Title/Abstract]) OR (“healthcare system“[Title/Abstract])) OR (“public health system“[Title/Abstract])) OR (“public healthcare system“[Title/Abstract])) AND (((((((institutionalization [Title/Abstract]) OR (institution [Title/Abstract]))) OR (“Institution Building“[Title/Abstract])) OR (“Institutional Based Trust“[Title/Abstract])) OR (“Social Institution“[Title/Abstract])) OR (“Institutional approach“[Title/Abstract]))	PubMed =((((((“health system“[Title/Abstract]) OR “healthcare system“[Title/Abstract]) OR “public health system“[Title/Abstract]) OR “public healthcare system“[Title/Abstract]) OR “public sector“[Title/Abstract])) AND ((((((institutionalization[Title/Abstract]) OR institution[Title/Abstract]) OR “Institution Building” [Title/Abstract]) OR “Institutional Based Trust” [Title/Abstract]) OR “Social Institution” [Title/Abstract])) AND (((governance[Title/Abstract]) OR “good governance“[Title/Abstract])) AND (“sustainable development” [Title/Abstract])
**Web of Science**	Web of Science = TITLE: (institutionalization OR institution OR “Institution Building” OR “Institutional Based Trust” OR “Social Institution” OR “Institutional approach”) AND TITLE: (“health system” OR “healthcare system” OR “public health system” OR “public healthcare system” OR “public sector”)	Web of Science = TITLE: (institutionalization OR institution OR “Institution Building” OR “Institutional Based Trust” OR “Social Institution”) AND TITLE: (“health system” OR “healthcare system” OR “public health system” OR “public healthcare system” OR “public sector”) AND TITLE: (governance OR “good governance”) AND TITLE: (“sustainable development”)
**Scopus**	Scopus = (TITLE-ABS-KEY (institutionalization OR institution OR “Institution Building” OR “Institutional Based Trust” OR “Social Institution” OR “Institutional approach”) AND TITLE-ABS-KEY (“health system” OR “healthcare system” OR “public health system” OR “public healthcare system”))	Scopus = (TITLE-ABS-KEY (institutionalization OR institution OR “Institution Building” OR “Institutional Based Trust” OR “Social Institution”) AND TITLE-ABS-KEY (“health system” OR “healthcare system” OR “public health system” OR “public healthcare system” OR “public sector”)) AND TITLE-ABS-KEY (governance OR “good governance”) AND TITLE-ABS-KEY (“sustainable development”)
**EMBASE**	(‘health system’:ab,ti OR ’healthcare system’:ab,ti OR ’public health system’:ab,ti OR ’public healthcare system’:ab,ti) AND (institutionalization: ab,ti OR institution: ab,ti OR ‘institution building’:ab,ti OR ‘institutional based trust’:ab,ti OR ‘social institution’:ab,ti OR ‘institutional approach’:ab,ti) AND (2000:py OR 2001:py OR 2002:py OR 2003:py OR 2004:py OR 2005:py OR 2006:py OR 2007:py OR 2008:py OR 2009:py OR 2010:py OR 2011:py OR 2012:py OR 2013:py OR 2014:py OR 2015:py OR 2016:py OR 2017:py OR 2018:py OR 2019:py OR 2020:py OR 2021:py)	(institutionalization: ab,ti OR institution :ab,ti OR ’Institution Building’:ab,ti OR ’Institutional Based Trust’:ab,ti OR ’Social Institution’:ab,ti) AND (‘health system’: ab,ti OR ‘healthcare system’: ab,ti OR ‘public health system’:ab,ti OR ‘public healthcare system’:ab,ti OR ‘public sector’:ab,ti) AND (governance: ab,ti OR ‘good governance’: ab,ti) AND (‘sustainable development’: ab,ti) AND (2000:py OR 2001:py OR 2002:py OR 2003:py OR 2004:py OR 2005:py OR 2006:py OR 2007:py OR 2008:py OR 2009:py OR 2010:py OR 2011:py OR 2012:py OR 2013:py OR 2014:py OR 2015:py OR 2016:py OR 2017:py OR 2018:py OR 2019:py OR 2020:py OR 2021:py)
**Google Scholar**	healthcare system + institutionalization + “Institutional approach” “Public health system” + institutionalization + “Institutional approach” “Public health services” + institutionalization + “Institutional approach”	healthcare system + institutionalization + “Institutional approach” + “Good Governance” + “sustainable development” “Public health system” + institutionalization + “Institutional approach” + “Good Governance” + “sustainable development” “Public health services” + institutionalization + “Institutional approach” + “Good Governance” + “sustainable development” healthcare system + institutionalization + “Institutional approach” + “Good Governance” + “sustainable health development” “Public health system” + institutionalization + “Institutional approach” + “Good Governance” + “sustainable health development” “Public health services” + institutionalization + “Institutional approach” + “Good Governance” + “sustainable health development”

#### Framework stage 3: selecting studies for inclusion

During the first phase, we identified 1061 studies (PubMed – 273; Web of Science – 77, Scopus – 200; EMBASE – 213 and Google Scholar – 298). The second phase revealed 463 studies (PubMed – 38; Web of Science – 143, Scopus – 259; EMBASE – 0 and Google Scholar – 23). Almost half of identified papers (N = 530; N = 231) were discarded as duplicates in each phase, respectively. We screened the titles and abstracts of 531 papers identified in phase 1 to mark ones that considered the concept of institutionalization in the health system, as well as their approach to explain the components of institutional approach. Besides, we screened titles and abstracts of 232 papers identified in phase 2 to identify those studied the relationship between institutionalization and good governance in the health system towards SHD. We continued to refine the inclusion/exclusion criteria until we reached the breadth of available evidence, as recommended by our adapted methodology [17].

**Fig. 1:**
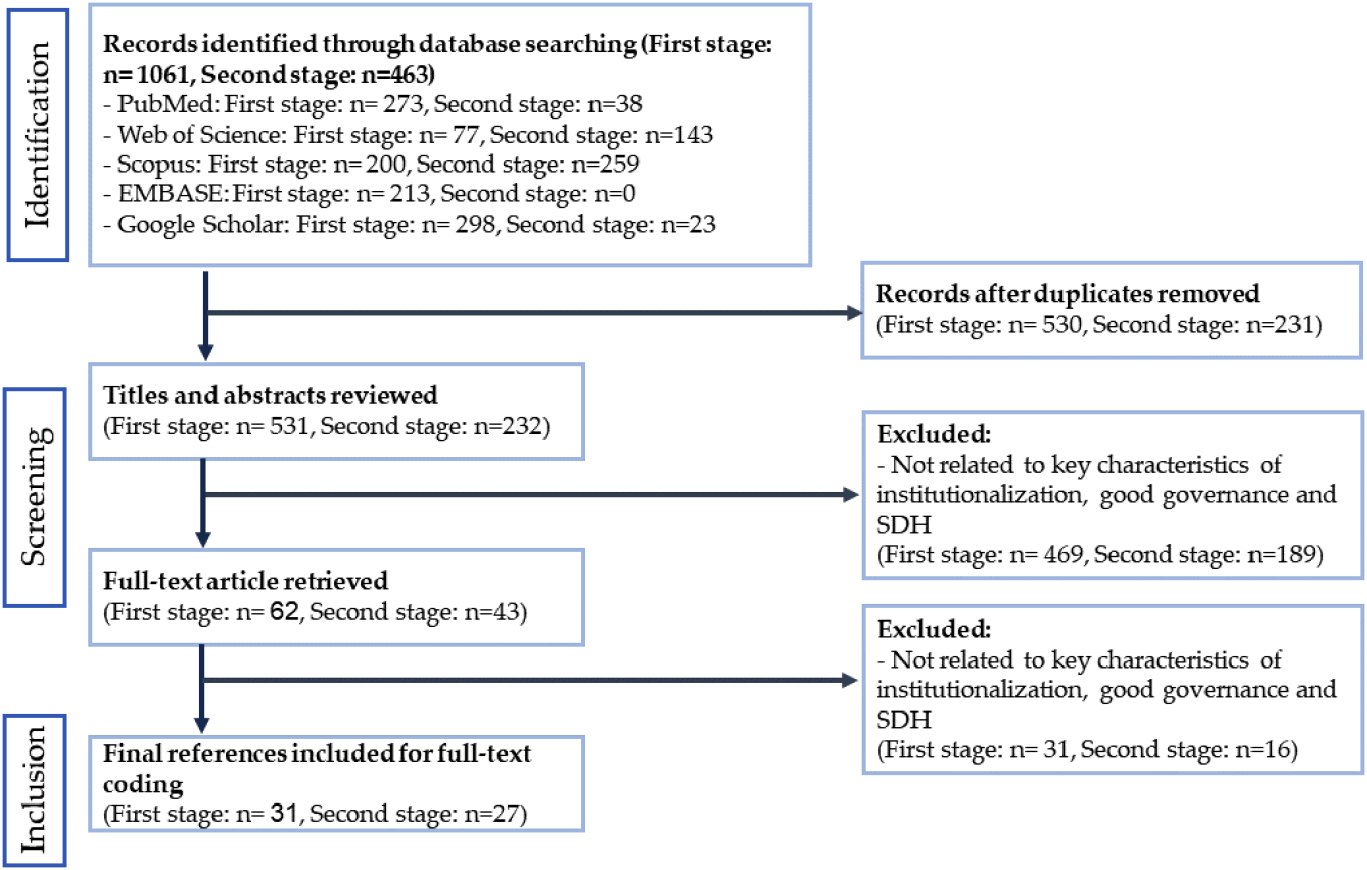
Literature search flow

This study only considered articles published in scientific journals after 2010. Studies in Persian and English were included. Papers that were not related to key characteristics of institutionalization, good governance, and SHD were removed at the title/ abstract screening stage and the full text of 62 and 43 papers of the first and second phase was further reviewed, respectively. Studies in the 1st phase demonstrated the application of the institutional approach in the health system in various countries, and those in the 2nd phase explored the relationship between achieving the institutional approach and having good governance. They examined the interconnection between each component of good governance and the elements of the institutional approach. In the 1st phase, we excluded studies that investigated the institutional approach establishment other system than health, including: (a) studies that explored the concept of institutionalization; (b) studies that described the institutional approach; (c) studies that investigated the institutional approach in any policy making system except the health system. In the 2nd phase, studies which did not consider the relationship between institutionalization and good governance in the health system were excluded, such as studies on economic, political, or cultural institutionalism. Finally, we included 31 and 27 studies from the 1st first and 2nd phases, respectively, on which we conducted further detailed analysis (Fig. 1).

#### Framework stage 4 and 5: data gathering, charting, organizing and summarizing

Using a purposive approach [16], the 1st author (HH) extracted and tabulated data from the accepted studies as follows: reference number, year of publication, first author’s name, context of study, type of study, aim and objectives for all studies related to both phases. To report findings, we explain the institutional concept, its components, and infrastructure, followed by describing the conceptual model of institutionalization in the health system.

### Innovation and limitations of the study

This study presents a novel framework for examining how good governance and sustainable health development can be fostered by institutionalization in various contexts. This framework could assist researchers and policy makers to comprehend the factors and processes that affect the adoption and performance of the institutional approach in the health system. The study could also enrich the literature on health system governance, which is a crucial topic for attaining universal health coverage and health security. However, the study may have neglected the complexity and diversity of the health system and its stakeholders. Furthermore, the institutional approach may not suit all settings and situations, and may encounter various difficulties and obstacles from political, economic, social, and cultural factors.

## Results

**Fig. 2:**
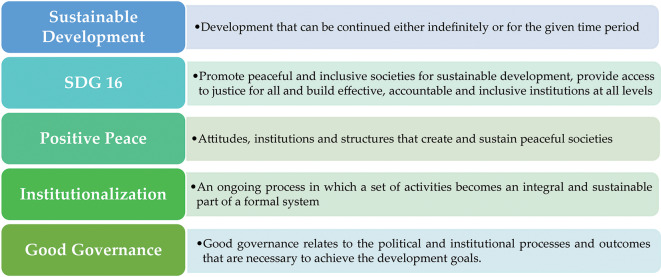
Definitions of main concepts [6, 21–24]

Figure 2 defines the key issues identified in our review, which are related to the concepts of SHD, its goals, pillars and principals; positive peace; good governance; components of institutional approach components, and their relations.

### Sustainable development

**Fig. 3:**
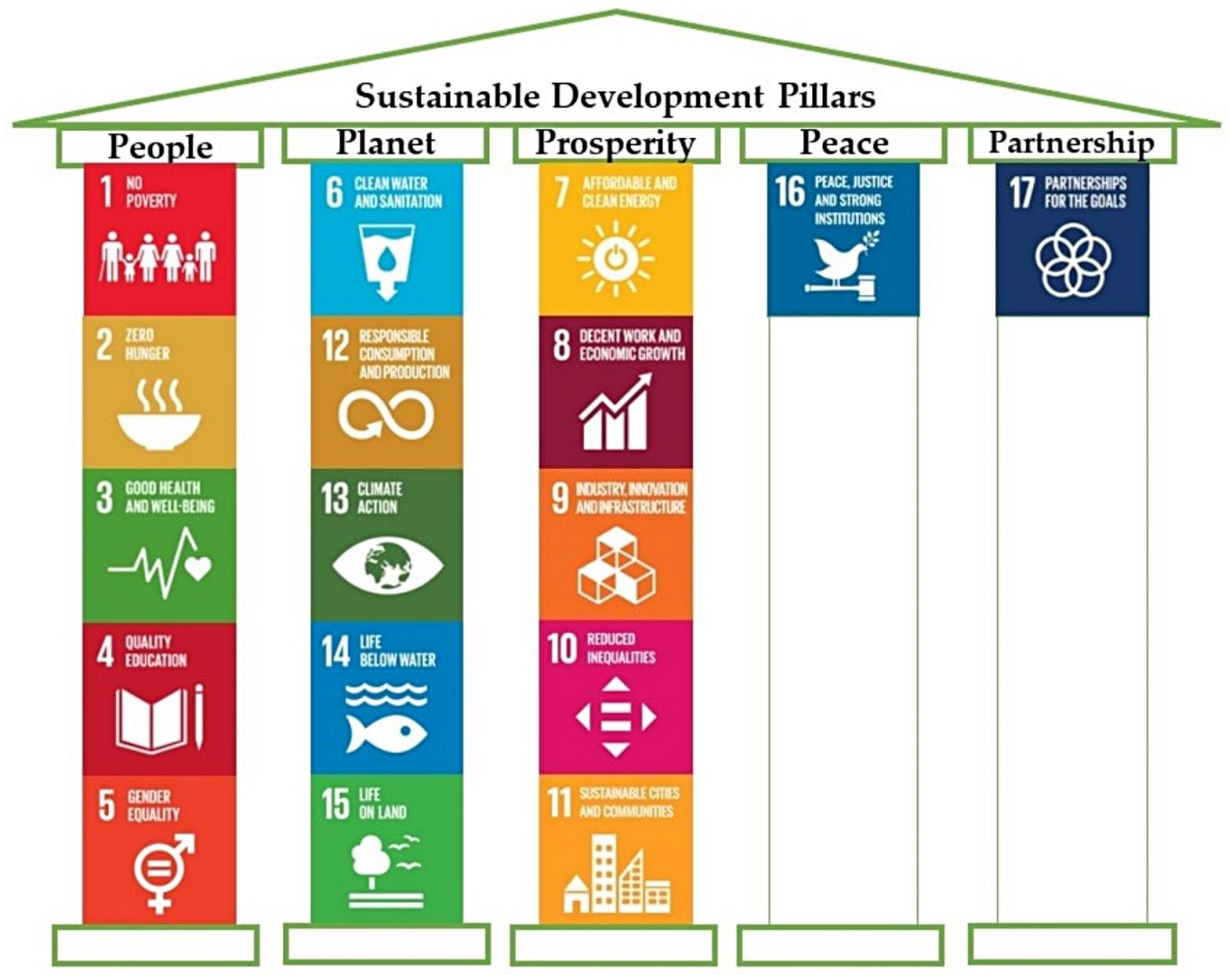
The pillars and its goals of sustainable development [26]

Development that can last forever or for the specified duration would be called sustainable development [25]. One of its most often cited definitions describes the concept as “development that meets the needs of the present without compromising the ability of future generations to meet their own needs” [22]. It allows society to engage with the environment in a way that does not endanger the resource for the future. In line with the underlying pillars of sustainable development i.e., people, planet, prosperity, partnership, and peace, the United Nations’ SDGs is an ambitious action plan to end poverty and put the world on a path to peace and prosperity based on the participation of all people in a healthy planet (Fig. 3). It aims to make the world a sustainable and resilient place for development over the next 15 years, through fostering a spirit of partnership among governments, the private sector, academia and civil society organizations [21, 22].

### SDG 16

**Fig. 4:**
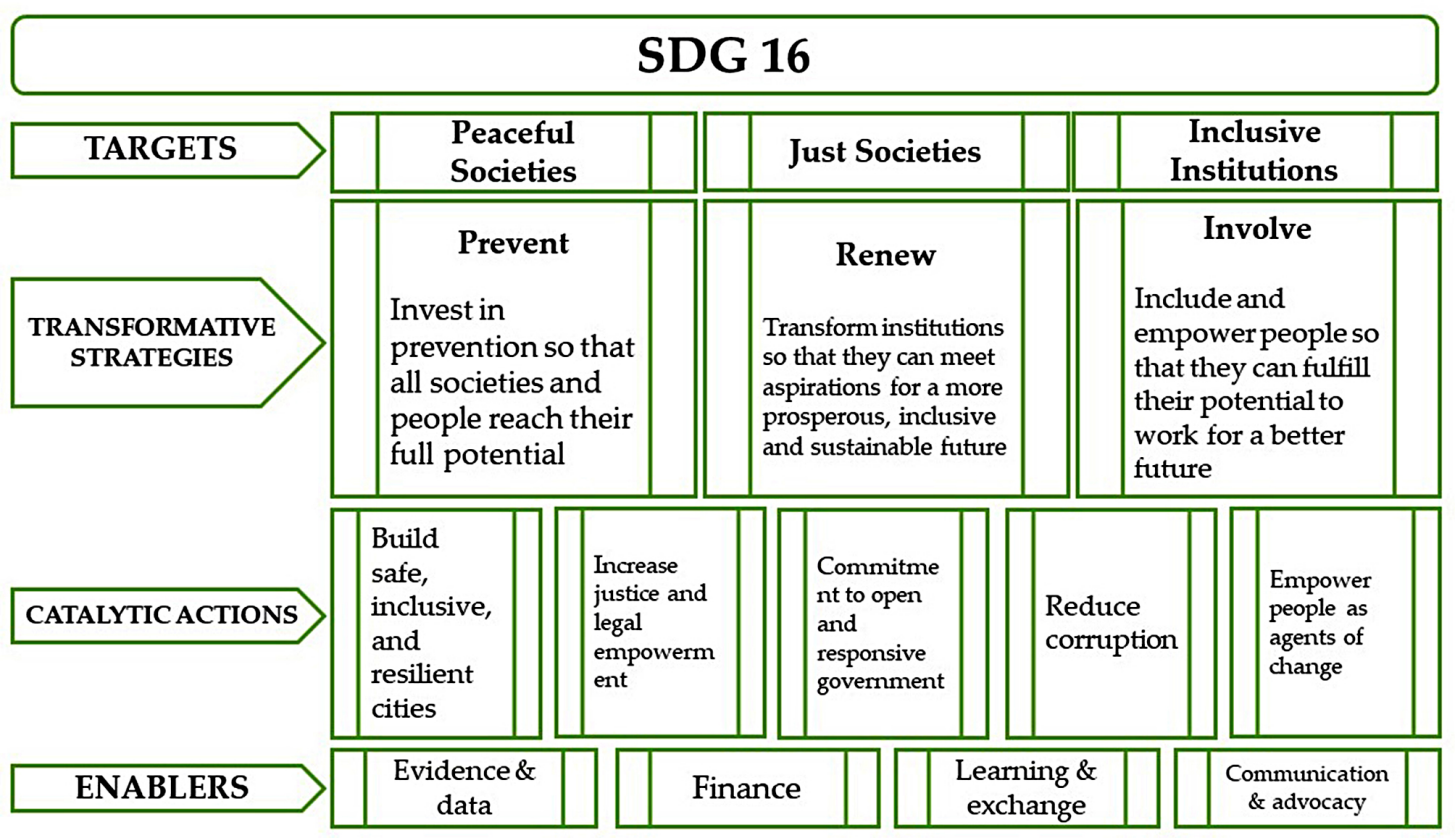
Concepts of SDG 16 [24]

SDGs’ goals and targets were set to end poverty, hunger, disease, and violence, protect the planet, and ensure prosperity for all citizens without any exception [27]. SDG 16 has declared the importance of promoting peaceful and inclusive societies, providing justice for all, and building effective, responsive and inclusive institutions at all levels to achieve sustainable development [21]. Achieving these targets requires adoption of some fundamental strategies, i.e., investing in prevention, renewing the institutions, and empowering people for an inclusive and sustainable future. As one of the most innovative aspects of the development framework, SDG 16 focus is on building safe and resilient cities, increasing justice, advancing government’s accountability, reducing corruption and empowering people (Fig. 4) [24].

There are two important concepts in this definition, peace and responsive institutions. As one of its five main pillars, achieving sustainable development would be impossible without peace [24]. Negative peace is the absence of direct violence and war, while positive peace is about the absence of indirect and structural violence such as exploitation, hunger, malnutrition, and corruption [28], which are essential for institutionalization. Furthermore, SDG 16 underpins the other sixteen SDGs, all of which also rest on institutions that are inclusive and capable of responding to the needs of the public transparently and accountably [24]. There is actually no one definition of ‘institution’. Rather, it is a universal agreement and constant patterns of responding to social needs, without frequent and short-term changes. Institutionalization has an effective role in maintaining human rights, environmental protection, stable economic conditions, and resources mobilization in providing basic services, which might lead to sustainable development in societies, eventually [21].

### Positive peace

Positive Peace is defined as “the attitudes, institutions and structures that create and sustain peaceful societies”. It can be used as the basis for measuring a country’s resilience - its ability to absorb, adapt and recover from shocks [29]. In the modern world, peace is a crucial component of sustainability and vice versa, especially in the context of rapid global changes. It is a precursor for achieving sustainability, particularly the sustainable use of resources, which is in turn associated with constructive conflict resolution [30].

Positive peace comprises eight pillars including: well-functioning government, sound business environment, equitable distribution of resources, acceptance of the rights of others, good relations with neighbors, free flow of information, high levels of human capital, and low levels of corruption, each of which represents a complex set of social dynamics that may lead to high levels of resilience and adaptability to change [23].

**Fig. 5:**
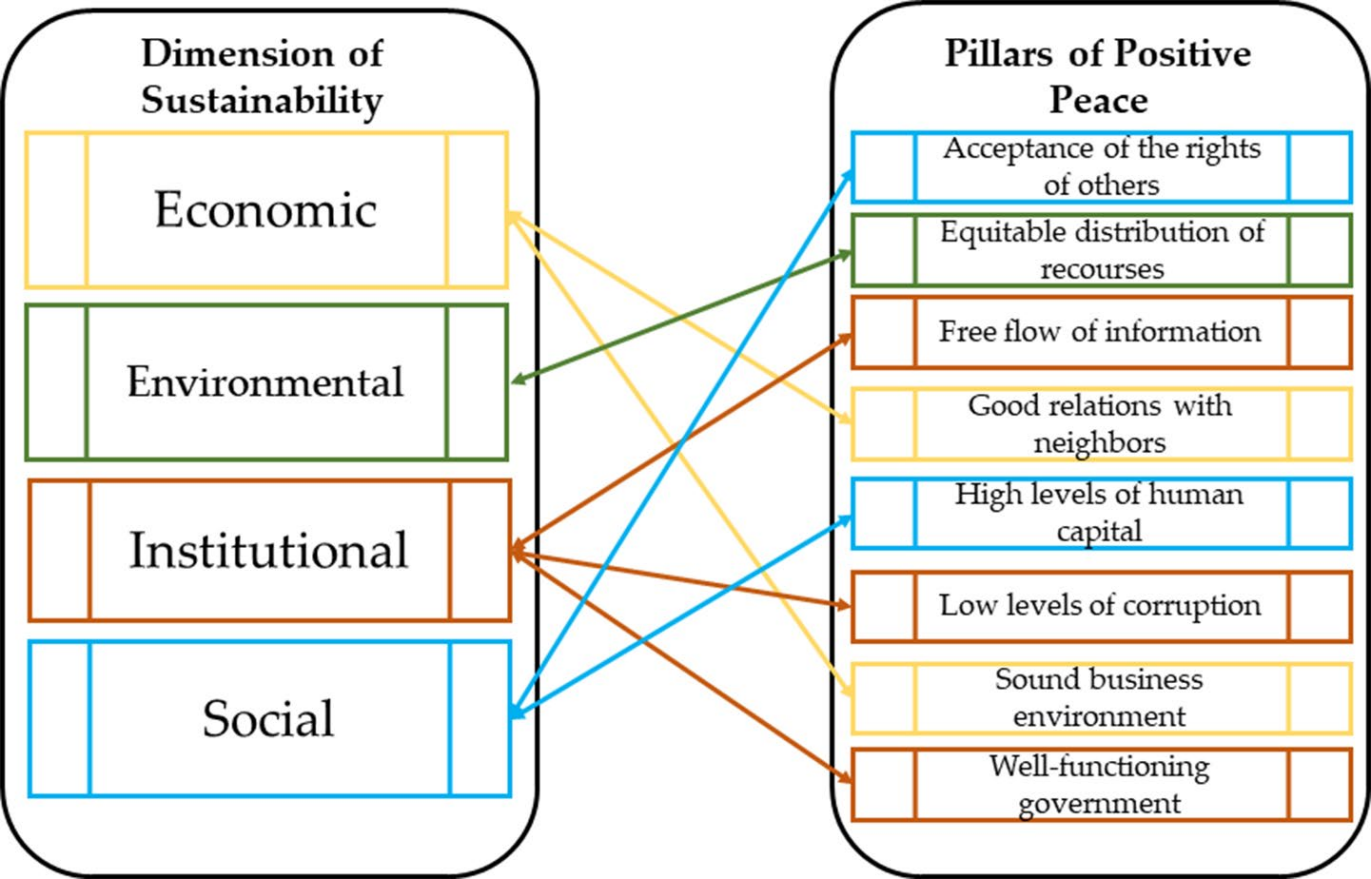
The relations between sustainability and positive peace [30]

Peace and sustainability are intricate notions with various meanings. Sustainability is also a vague notion and as value-laden as peace. The nexus of peace and sustainability has received growing interest in recent decades. Figure 5 shows the positive relationship between the dimensions of sustainability and the pillars of peace. The environmental aspect of sustainability, emphasizes the equitable allocation of resources [30]. Conflicts have immediate adverse impact on the environment. For example, warring parties may exploit scarce natural resources for detrimental purposes, such as funding their activities, thereby exacerbating the conflict. Furthermore, inappropriate use of natural resources can lead to inequitable allocation and access, which is a major cause of many conflicts, such as the one in South Sudan [31].

The social aspect addresses matters concerning high human capital (through learning and other types of social interactions) and respect for the rights of others (especially of disadvantaged groups in society) [30]. Social sustainability refers to social processes that foster social capital and well-being intra/intergenerational. The ecosystem’s deterioration undermines its provision of resources and services, which may consequently imposes stress on the people who depend on them and their social relations. The ecosystem and human well-being are mutually dependent, such that preserving ecological well-being is vital to avoid poverty. Environmental change has a greater impact on the poor and the populations affected by conflict [32].

The economic aspect requires good connections with neighbors (particularly inter-state economic and political partnership) and a stable business environment (in terms of harmonizing business interests with peace and sustainability aims) [30]. Peace and conflict prevention are crucial for economic development. In a globalized economy, the private sector can play a role in, and also gain from, peace. Multinational enterprises participate in peacebuilding activities to mitigate investment risks in post-conflict societies, which then improves their profitability and competitive advantage [33]. On the contrary, some argue that the globalized economy has adversely affected the environment and eroded social capital by interrupting local community practices and interactions. Multinational corporations are mainly concerned with maximizing benefits rather than pursuing altruistic and normative goals [34].

Lastly, the institutional aspect shows the significance of democratic processes that include effective government, low corruption, and free information exchange [30]. A well-functioning government that operates democratically and effectively is best suited to cooperate with international partners to execute peace and sustainability projects due to its political legitimacy and capacity for institutional reform [35].

 [31]Among these pillars, effective government has been recognized as interrelated with achieving positive peace. Positive peace not only provides the favorable conditions for achieving good governance, but also it has been the result of good governance being established [36]. Governance systems promote peace when they are inclusive, participatory, and accountable, and have the capacity to provide a wide range of public goods. In this regard, increasing peace results in spending more on both education and health [37]. Consequently, since health and peace are closely related and both are basic human rights, one cannot exist without the other [38].

### Institutionalization

**Fig. 6:**
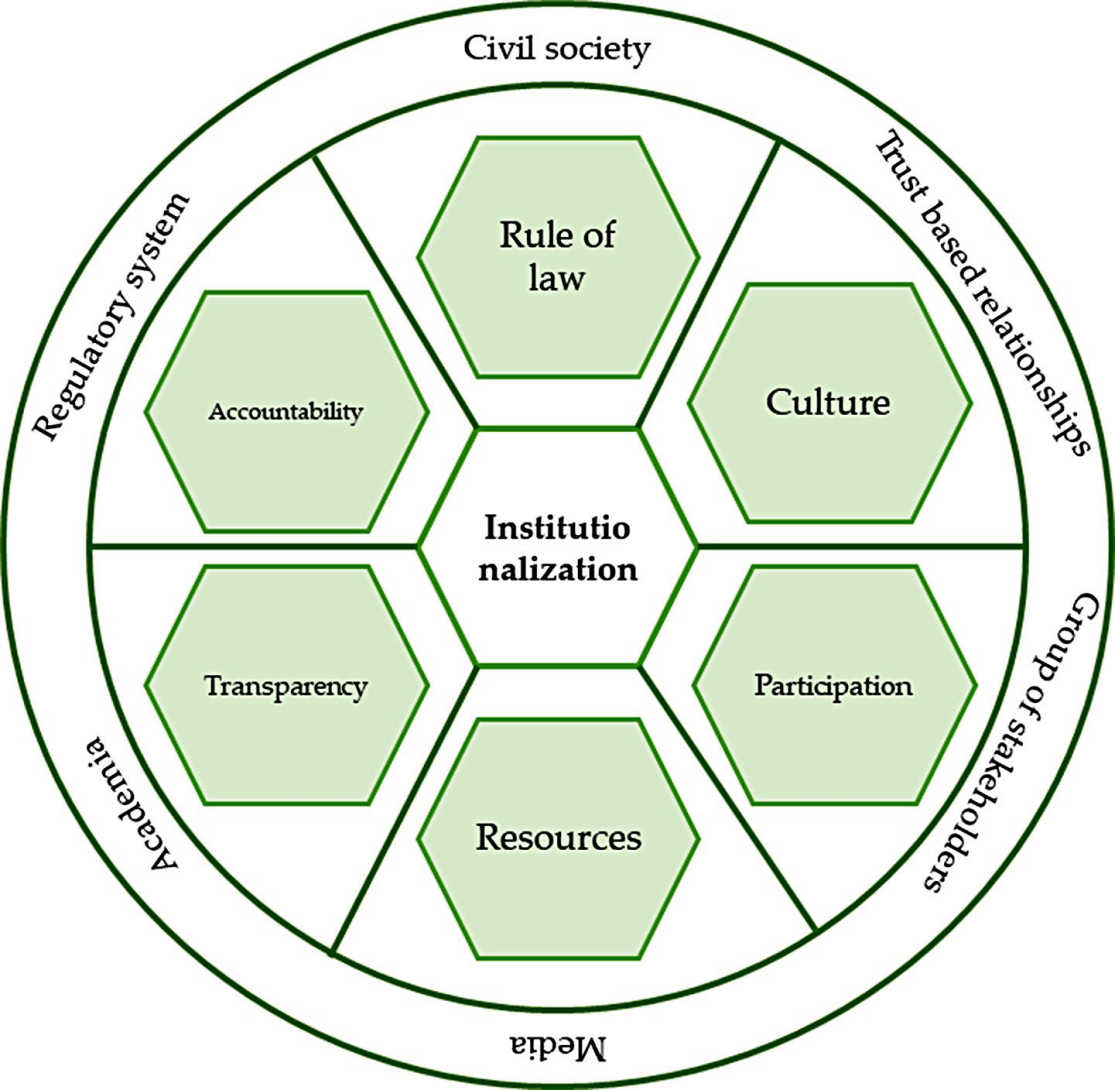
Elements of institutionalization (Source: Authors)

Institutions can have a large influence in forming policy choices, involving stakeholders and conveying decisions [39]. They specify who has the right to act and to make decisions that affect everyone, they make actors’ actions consistent and transparent, and connect those who have power to those who are impacted by decisions [40]. Institutionalization, from this viewpoint, is possibly a very efficient way of regulation. It establishes steadiness and order in defined fields, without requiring frequent authoritative actions [1]. It is a lasting process in which a set of actions becomes a vital and sustainable part of a formal system, and “alters the organization in a stable way” so that its parts are fully assimilated into standard practice and used over time [41]. It depends on the involvement of senior politicians specifically, but also demands the active contribution of other stakeholders at all levels of the political and administrative systems, and of development partners [42]. Reaching institutionalization concept, in many ways, shows the efficiency of governance, and is one of the important means of creating good governance. This mechanism is often marked by major features which ensures that law is followed, the perspective of all stakeholders is considered, the voice of civil society are listened, strong regulatory system is regarded, and inclusion is achieved (Fig. 6) [4].

### Good governance

**Fig. 7:**
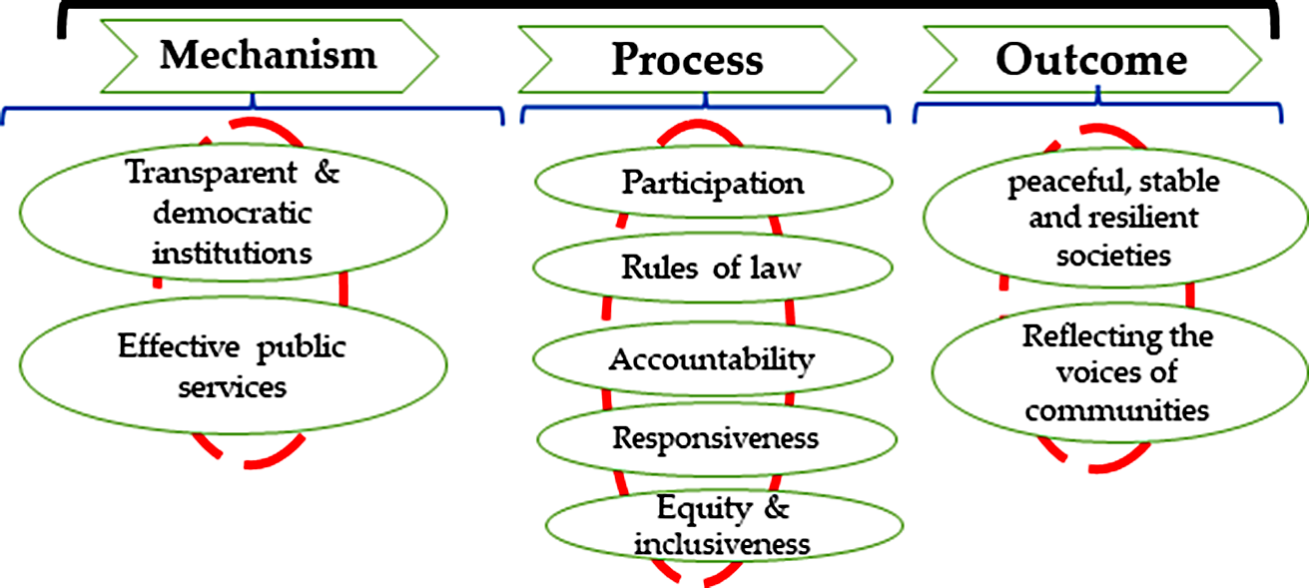
Indicators of good governance (Source: Authors)

Good governance broadly relates to institutional matters, social fairness and inclusiveness. It is linked to “a set of qualitative features relating to processes of rulemaking and their institutional bases”. It embodies values such as increased participation, transparency, responsibility, and public access to suitable information. It also assists to fight corruption and protect both fundamental human rights and the rule of law [43]. The sustainable development agenda is evidently dedicated to good governance and its essential role. Goal 16 indicates “effective governance institutions and systems that are responsive to public needs deliver essential services and promote inclusive growth”. Institutions are the basics for good governance. Besides, good governance includes relations between state and people. To put it briefly, good governance can be attained by applying institutionalization approach, which helps governments to perform better, more responsibly and more efficiently in dealing with development programs, and also establishes a favorable environment for sustainable development mechanisms to operate. The quality of participation is essential for governance processes, which means that the political, social and economic priorities are based on a wide agreement in society and that the decision-making process listens to the voices of the excluded, poorest and most vulnerable [21]. Rule of law as another good governance principle entails several elements, such as judicial independence, legal equality of citizens, and the entitlement of citizens to pursue legal actions against their governments [44]. Rule of law is strong when people support it not out of fear but because they benefit from its effectiveness. It requires the cooperation of state and society, and is based on complex and deeply rooted social processes, not just legal penalties and sanctions [45]. Accountability is a key component of good governance, which implies the obligation to answer for decisions or actions. It depends on institutional design and political energy, and requires the participation of various actors in demanding and providing explanations. Accountability also entails the assurance of safety, honesty, and responsiveness for those who seek and offer accountability [45] Good governance can result in societies that exhibit characteristics of peace, stability and resilience, where the provision of services is aligned with the demands of the communities, incorporating the perspectives of the most disadvantaged and marginalized groups (Fig. 7) [21] [21].

### Conceptual framework of institutionalization in health

**Fig. 8:**
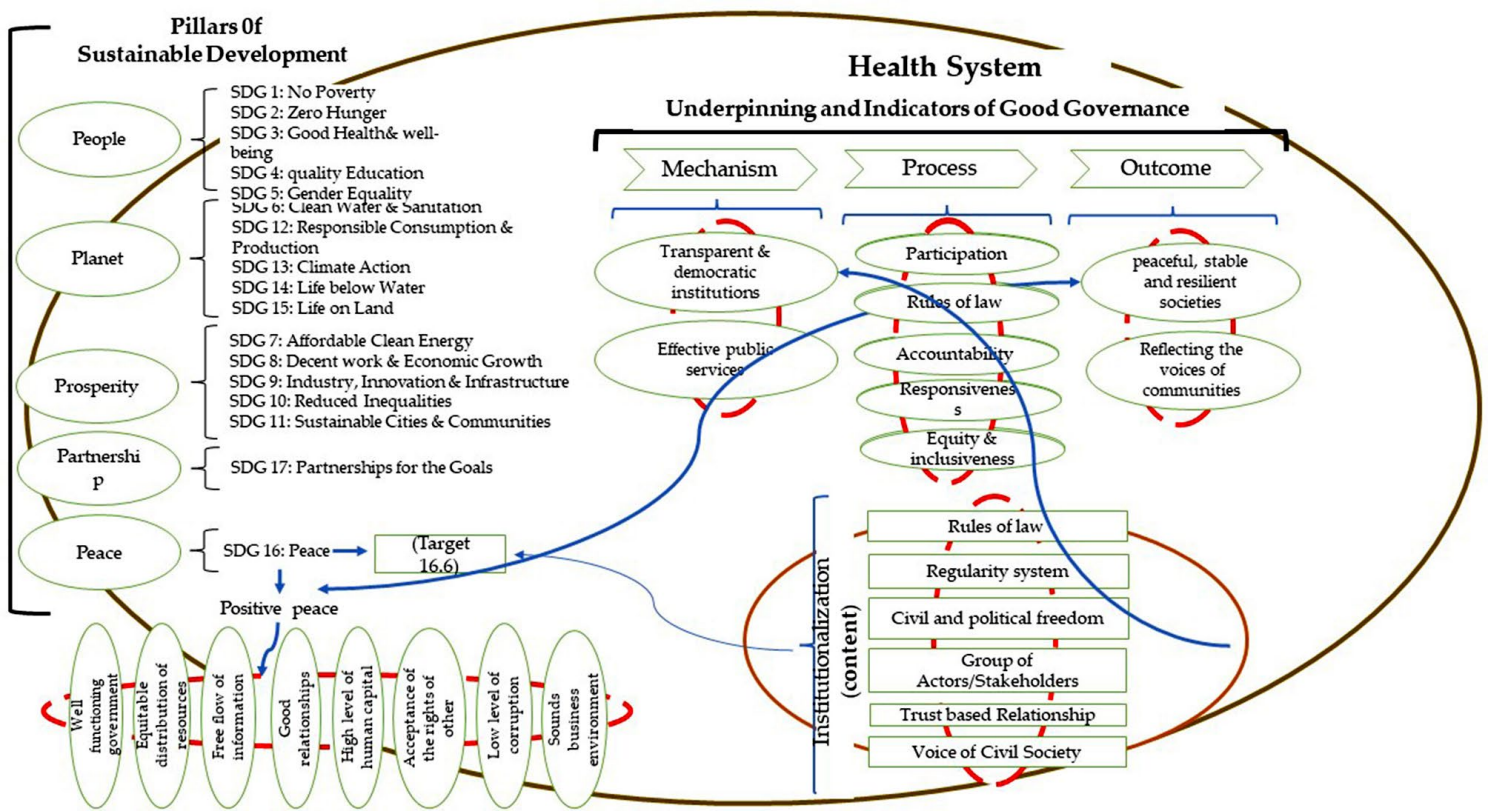
The institutionalization framework (Source: Authors)

Governments, by effective decision-making based on accountability, transparency, and participation, are responsible to attain sustainable development and make tangible improvements to their citizens’ quality of life and well-being. Appropriate and contextual institutionalization is the key, we advocate, to reach sustainable development in any setting. Institutionalization in health refers to the process of establishing and embedding health policies, programs, and practices within a system, organization, or community. Institutionalization can enhance the sustainability, effectiveness, and efficiency of health interventions, as well as the accountability and responsiveness of health actors. To achieve institutionalization in health, various factors need to be considered, such as the political, economic, social, and cultural context, the stakeholders’ interests and power relations, the evidence base and innovation potential, and the monitoring and evaluation mechanisms [6]. Figure 8 summarizes our findings, in a format of a proposed conceptual framework, to underpin the role of institutionalization in the health system to achieve sustainable development. This conceptual framework presents all the main components examined in this study and their interrelations within the health system context. To develop this framework, all the sustainable development goals and their connections with its five pillars were illustrated. The research question is derived from the 16th Sustainable Development Goal (SDG 16), which pertains to peace. The concept of positive peace was derived from SDG 16 and its eight components were demonstrated. Having accountable and transparent institutions is one of the targets of SDG 16, which is the foundation and relevance of this paper for achieving it in the health system, aiming to reach sustainable health development. Good governance requires transparent and democratic institutions and an institutional approach as the means to achieving it, while sustainable and resilient societies, where all groups participate extensively in decision-making are the ends of implementing good governance [4]. Peace and sustainable development are the objectives of good governance, which necessitates the presence of good governance as a precondition for their attainment [46].

## Conclusion

In this scoping review, we analyzed the findings of selected published studies that describe the concept of institutional approach and its components in the health system, its relationship with good governance and their play in reaching sustainable health development. Our proposed conceptual framework introduces the identified elements and their relationship, which are crucial for institutionalization. Our framework has depicted, we envisage, the relationship between successful implementation of institutionalization components to reach sustainable development.

As a cross-cutting concept which accommodates positive peace and good governance, institutionalization cannot be considered in an isolated context. Hence, the need for leaders and senior politicians to initiate the process of institutionalization at the national level, while fostering all prerequisites and right conditions, i.e., effective parliamentary scrutiny, a functioning and active civil society throughout their communities. Societies with meaningful public participation in decision-making, sustainable livelihoods, justice, human rights, transparency, accountability and respect for the rule of law are far more likely to reach institutionalization. As COVID-19 has slowed down the global pathway towards SDGs, as its goals and targets might be unlikely to achieve by 2030, greater investment on institutionalization, particularly in the LMICs is fundamental, now more than ever, to make human societies work and practice for sustainable development.

### Proposed future research

Researchers can use our finding to generate research questions to be addressed by systematic reviews in the future. Some possible research questions are:

- What are the effects of institutionalization for good governance on health outcomes and health equity in different contexts and settings?
- What are the best practices and strategies for implementing institutionalization for good governance in health systems and organizations?
- What are the barriers to and facilitators for institutionalization of good governance in health policy and decision-making processes?
- How can institutionalization for good governance be measured and evaluated in health systems and organizations?

## Data Availability

Not applicable.
